# Fecal Metabolomics Reveals Distinct Profiles of Kidney Transplant Recipients and Healthy Controls

**DOI:** 10.3390/diagnostics11050807

**Published:** 2021-04-29

**Authors:** Soumaya Kouidhi, Oumaima Zidi, Muhanad Alhujaily, Nessrine Souai, Amor Mosbah, Tareg M. Belali, Kais Ghedira, Imene El Kossai, Jamelddine El Manaa, Wissem Mnif, Ameur Cherif

**Affiliations:** 1 Laboratory of Biotechnology and Valorisation of Bio-GeoRessources, Higher Institute of Biotechnology of Sidi Thabet, BiotechPole of Sidi Thabet, University of Manouba, Ariana 2020, Tunisia; oumaima.zidi@hotmail.fr (O.Z.); nessrine.souai@fst.utm.tn (N.S.); amor.mosbah@gmail.com (A.M.); ameur.cherif@uma.tn (A.C.); 2 Department of Clinical Laboratory, College of Applied Medicine, University of Bisha, P.O. Box 551, Bisha 61922, Saudi Arabia; malhujaily@ub.edu.sa; 3 Faculty of Applied Medical Sciences, University of Bisha, 255, Al Nakhil, Bisha 67714, Saudi Arabia; blaly@ub.edu.sa; 4 Laboratory of Bioinformatics, bioMathematics, and Biostatistics (LR16IPT09), Pasteur Institute of Tunisia, University of Tunis El Manar, Tunis 1002, Tunisia; ghedirakais@gmail.com; 5 Unit of Organ Transplant, Military Training Hospital, Tunis 1008, Tunisia; imenelkossai@gmail.com (I.E.K.); jamel_manaa@yahoo.fr (J.E.M.); 6 Department of Chemistry, Faculty of Sciences and Arts in Balgarn, University of Bisha, P.O. Box 199, Bisha 61922, Saudi Arabia; 7 Institut SupéRieur de Biotechnologie Sidi Thabet, University of Manouba, BVBGR-LR11ES31, Biotechpole Sidi Thabet, Ariana 2020, Tunisia

**Keywords:** fecal metabolome, kidney transplantation, microbiota, biomarkers

## Abstract

Monitoring graft recipients remains dependent on traditional biomarkers and old technologies lacking specificity, sensitivity, or accuracy. Recently, metabolomics is becoming a promising approach that may offer to kidney transplants a more effective and specific monitoring. Furthermore, emerging evidence suggested a fundamental role of gut microbiota as an important determinant of patients’ metabolomes. In the current study, we enrolled forty stable renal allografts recipients compared to twenty healthy individuals. Samples were taken at different time points from patient to patient following transplantation surgery, which varied from 3 months to 22 years post-graft. All patients started the immunosuppression therapy immediately following kidney graft (Day 0). Gas chromatography–mass spectrometry (GC–MS) was employed to perform untargeted analysis of fecal metabolites. Globally, the fecal metabolic signature was significantly different between kidney transplants and the control group. Fecal metabolome was dominated by lipids (sterols and fatty acids) in the stable transplant group compared to the controls (*p* < 0.05). Overall, 18 metabolites were significantly altered within kidney transplant recipients. Furthermore, the most notable altered metabolic pathways in kidney transplants include ubiquinone and other terpenoid-quinone biosynthesis, tyrosine metabolism, tryptophan biosynthesis, and primary bile acid biosynthesis. Fecal metabolites could effectively distinguish stable transplant recipients from controls, supporting the potential utility of metabolomics in rapid and non-invasive diagnosis to produce relevant biomarkers and to help clinicians in monitoring kidney transplants. Further investigations are needed to clarify the physiological relevance of fecal metabolome and to assess the impact of microbiota modulation.

## 1. Introduction

Chronic kidney disease (CKD) is a prevalent and chronic life-threatening disease marked by socioeconomic impact as well by high morbidity and mortality [1]. Due to the high complexity of its clinical management, 2–3% of the total healthcare expenditure in developed countries is directed to treating patients with end-stage renal disease [2]. For instance, kidney transplantation is a long-term treatment for CKD. However, it is closely correlated with poor outcomes of rejection and the development of side risk factors, such as dyslipidemia, diabetes, obesity, hypertension, and chronic viral infections (Hepatitis B Virus (HBV), Cytomegalovirus (CMV), or Betapolyomavirus (BK virus)), or immunosuppressive nephrotoxicity. Yet, there are still doubts about the markers that can predict a better graft function or promptly detect a kidney rejection. The current clinical measurements are simplistic, i.e., the used biomarkers, such as serum creatinine or urinary albumin, blood pressure, or blood glucose, lack sensitivity and specificity. First, it has been shown that serum creatinine lacks a high predictive value. In fact, increased serum creatinine was reported only when 40–50% of the renal parenchyma was damaged [3]. This may lead to the lack of detection of early stages of acute or chronic kidney failure and, therefore, to the delayed application of detailed diagnostics and the implementation of therapeutic interventions [4]. Second, although it has been established that microalbuminuria could indicate kidney damage, considerable doubt has emerged that it is a predictor of diabetic nephropathy [5]. Therefore, there is a need for more sensitive and specific biomarkers that can predict a patient’s kidney function. Although renal biopsy remains the gold standard by which essential diagnostic and prognostic information is obtained after kidney transplantation, recent studies suggest that even the “gold-standard” histology assays could be problematic [6]. Therefore, considering the limitations of routinely used methods in the clinical setting, new analytical tools are essential to find robust biomarkers for the right prediction of the graft function and/or renal side effects of calcineurin inhibitors in kidney transplantation. The ideal biomarkers are those that can be detected easily and non-invasively to distinguish patient subgroups [7]. In this clinical context, metabolomics is a high-throughput measurement and analysis of metabolites. It has emerged as a promising method for the identification of metabolic signatures related to diseases. By covering a huge spectrum of metabolites, metabolomics aims to draw a representative picture of a biological system and more closely reflects the dynamic phenotype [8]. Therefore, metabolomics is an exciting approach to find key biomarkers that may contribute to more effective and specific disease monitoring. However, its clinical application in the context of kidney transplantation is still very much in its infancy. Recent advances have recognized that metabolomics studies that have been performed on the serum and urine samples of patients suffering from kidney diseases may offer a predictable explanation for kidney function, kidney injury, and immunosuppressive drug toxicity [9,10]. Accordingly, earlier reports have identified altered levels of several classes of metabolites that have been recognized as crucial markers to delineate renal stress or dysfunction [11]. One major concern that has emerged from the recent findings is the contribution of the gut microbiome to the metabolome signature. Several gut-derived metabolites were previously described to be linked to kidney disorders, including indoxyl sulfate, p-cresol sulfate, and trimethylamine-N-oxide (TMAO) [12,13]. Nevertheless, dysbiosis of gut microbiota may aggravate the clinical outcomes in kidney allograft patients. Despite the important role played by host gut microbiota to modulate the metabolic profile, the mechanisms underlying this interaction remain to be fully investigated.

Several research studies have reported a metabolomic comparison between kidney graft recipients and healthy controls to assess the shift in certain metabolic pathways [14]. Hence, all the earlier studies that have successfully reported the application of metabolomics to kidney transplantation have mainly explored urine, plasma, or serum samples, but never fecal samples. Therefore, in this pioneering study, we recruited stable kidney graft recipients; samples were taken at different time points from patient to patient following transplantation surgery. We divided our cohort into subgroups depending on the post-graft period, short post-graft period (“SG”: from 3 months to 1 year; n = 11), medium-length post-graft period (“MG” from 1 year to 10 years; n = 20), and long post-transplant period (“LG” from 10 to 22 years n = 9). See data in Table S1. All patients started immunosuppression therapy immediately following kidney graft (Day 0). We performed the metabolomics approach using the GC–MS platform to explore the changes in metabolites in the feces of renal transplant patients. We aimed to 1: identify and characterize specific fecal metabolite profiles in stable kidney transplants following immunosuppressive therapy compared to healthy subjects; 2: provide novel metabolic biomarkers to gain further insights into the crosstalk underlying the gut–kidney axis; 3: establish a non-invasive, more sensitive, and specific method to complement existing ones to improve current diagnostic standards and the monitoring of kidney transplants that may prevent organ rejection.

## 2. Materials and Methods

### 2.1. Samples Collection

Fecal specimens from (n = 40) stable kidney allograft recipients were collected from the organ transplantation unit at the Military Hospital of Tunis. Samples were taken at different time points from patient to patient following transplantation surgery. We divided our cohort into subgroups depending on the post-graft period, short post-graft period (“SG”: from 3 months to 1 year; n = 11), medium-length post-graft period (“MG” from 1 year to 10 years; n = 20), and long post-transplant period (“LG” from 10 to 22 years n = 9). We conducted further analysis of the fecal specimens based on the health status of the participants and whether or not they suffered from any lifestyle diseases that were named associated diseases and labelled as (AD). In the present study, n = 24 patients suffered from one or multiple complications from the following list: obesity, diabetes, high blood pressure, and dyslipidemia. Furthermore, n = 16 were kidney graft recipients that did not suffer from any associated diseases (labeled No_AD group). It is worth noting that the primary diseases factor was also investigated but not reported in the present study due to non-significance. This could be due to subjects covering a broad range of nephropathies. See data in Table S1. All patients started immunosuppression therapy immediately following kidney graft (Day 0). Healthy volunteers (n = 20) provided control samples to compare healthy and patient gut metabolomes. Each individual in the above-mentioned cohort signed an informed consent form approving their participation in the present trial and the sharing of clinical data for research purposes. Samples were collected in sterile stool specimen containers, immediately delivered in pre-cooled boxes, and stored at −80 °C until metabolite profiling was performed. All subjects gave their informed consent for inclusion before they participated in the study. The study was conducted in accordance with the Declaration of Helsinki, and the protocol was approved on 5 March 2018, by the Ethics Committee of the Military Hospital of Tunis N°05032018. Clinical and demographic data of study groups are further detailed in Supplementary Table S1.

### 2.2. GC–MS Sample Preparation and Metabolites Extraction

Fecal water was extracted by performing a successive two-solvent (ethanol and Diethyl ether)-based metabolite extraction protocol. It has been reported in a recent study that extraction efficiency was high for ethanol or methanol and isopropanol protocols [15,16]. As methanol is more carcinogenic than ethanol, we chose to use ethanol as an extraction solvent. Fecal samples were thawed, and 3 g of each stool specimen was mixed with pre-cooled ethanol (−20 °C) in the ratio of 3:20 (g:mL; feces: ethanol). The mixtures were homogenized by sonication for 30 min and centrifuged at 4000 rpm for 20 min at 4 °C. The supernatants were then transferred and filtered through a 0.45 µm Millex-GV Syringe Filter. A second extraction was conducted by adding 20 mL of Diethyl ether to the pellets. The mixtures were well shaken, vortexed for 3 min, centrifuged at 4000 rpm for 20 min at 4 °C, and then filtered through a 0.45 µm Millex-GV Syringe Filter. All filtered fecal waters were divided into a 150 µL aliquot per Eppendorf tube and dried to the complete dryness of solvents, under reduced pressure in a speed vacuum at 10 °C, to obtain a pellet of concentrated metabolites. All the extracted metabolites were stored at −20 °C until analysis. For the first step, 2 mg ± 0.01 of each sample pellet was grated and diluted with 500 µL of extraction solvent. The mixtures were filtered through a 0.45 µm Millex-GV Syringe Filter. All the samples were run on GC–MS with a 500 µL blank of each extraction solvent. The second step was the derivatization: 2 mg ± 0.01 of each sample was derivatized by adding 800 µL of N-Hexane and 400 µL of (1 M) Sodium methylate to the metabolite pellets. The resulting solution was then vortexed, 200 µL of H_2_SO_4_ (0.1 M) was added and the mixture was homogenized. An amount of 500 µL of the supernatants was transferred to GC–MS glass vials. A blank with MilliQ water was prepared and treated the same as the derivatized samples. It is important to note that exactly the same protocol was applied for all the tested samples. A triplicate was performed for every sample to validate the obtained profile. We assessed relative and absolute abundance of chromatographic peaks in quality control samples as described previously [17]. Malathion and chlorpyrifos were used as internal standards. Briefly, we used a six-point calibration curve of a mixture of quality control reference standards, in addition to method blanks and reagent blanks, for each new batch of analyses starting from the lowest concentration to the highest.

### 2.3. GC–MS Analysis and Metabolites Detection

The samples were analyzed using the Agilent GC 7890B. MS 240 ion trap Gas Chromatography technology equipped with an MS detector (GC–MS) (Agilent, CA, United States). Injections were in splitless mode for 0.75 min, using a 2 mm I.D. non-deactivated direct liner. The separation was carried out on a HP-5MS capillary column (30 m × 0.250 mm; 0.25 μm film thickness) (Agilent, CA, United States). The analysis was carried in full scan mode for 60 min. An autosampler injected 1 µl of each sample, and the separation was performed using the column in split mode and ionization range from 50 to 1000 mV. The carrier gas was helium with a flow rate of 1.1 mL/min. The injector temperature was set at 280 °C, and GC oven temperature was programmed at 40 °C for 2 min; then, a slope at 50 up to 250 °C was maintained for 20 min. The analysis was carried in full scan mode for 60 min.

### 2.4. Identification and Comparison of Volatile Compounds

Mass spectral data processing and metabolite identification were performed using an Automated Mass Spectral Deconvolution and Identification System (AMDIS) (AMDIS-version 2.71, 2012) and the National Institute of Standards and Technology (NIST) (version 2.0, 2011) database. The detected metabolite peaks were identified using three components within NIST; these were a match of > 800, a 90% probability of a match to NIST library standards, and a head-to-tail comparison of the fragments. Metabolite validation was performed by matching experimental tandem MS spectra, retention time, and cas number of the metabolic features against PubChem library (https://pubchem.ncbi.nlm.nih.gov/) (accessed on 1 January 2004) as well as the spectral database Human Metabolomic Data Base (HMDB) (https://hmdb.ca/) (accessed on 1 January 2007). A compound was considered to be present when it satisfied these 3 criteria. This process provides a relative ion abundance; no units of ion abundance are available. A compound with a similarity index more than 80% was considered as a potential biomarker; therefore, compounds that were found in less than 20% of the entire sample cohort were removed from further analysis [18].

### 2.5. Data Analysis

We attempted to analyze metabolic profiling within the two groups by performing multivariate statistical analysis using SIMCA 16. Initially, the principal component analysis (PCA) was carried out to identify any outliers within the data set. Then, an orthogonal partial least squares-discriminant analysis (OPLS-DA) was applied to optimize the separation between the different groups. The model robustness was evaluated with the R2Y (fraction of variance), the Q2 (model predictability), and *p*-values. Close to 1, R2Y and Q2 values indicate an excellent model, whereas low values are indicative of model over-fitting. The statistical model was tested for robustness by a Y-permutation performed by PLS-DA, which confirmed the observed metabolic variations. The statistical model was tested for robustness by a Y-permutation performed on PLS-DA, which confirmed the observed metabolic variations, and by the use of a CV-ANOVA from SIMCA-P 16 (analysis of variance in the cross-validated residuals of a Y variable). A hierarchical cluster analysis heat map was obtained using the ward clustering algorithm and Euclidean distance calculation to further confirm the results of PLS-DA and to show the distribution of metabolites among all individuals. Pathway analysis was performed using metaboanalyst4.0.

### 2.6. Selection of Biomarkers

We constructed Receiver Operating Characteristic (ROC) curves to check the accuracy of the model, using metaboanalyst v 4.0. A forward stepwise logistic regression model was constructed to design the best metabolite combination. ROC curves were used to test the accuracy of the model. The global performance of each biomarker was evaluated using the Area Under the Curve (AUC) and the determination of sensitivity and specificity [19]. The data obtained were subjected to an unpaired non-parametric test (Wilcoxon rank-sum test, also known as the Mann–Whitney U-test) within MetaboAnalyst, and false discovery rates (FDR) were calculated by the Significant Analysis of Microarray (SAM) analysis, which is essentially used for microarray data but also used for metabolomic data (GC–MS; LC-MS and NMR compounds), to discover if the metabolites were significantly different between groups [20]. All features with FDR values below 0.05 indicated that these features can indeed be regarded as potential “biomarkers”. A metabolomic pathway analysis (MetPA) [21] was applied, by Metaboanalyst v 4.0 (accessed on 1 January 2009), to the selected biomarkers to find the most relevant pathways involved in renal transplantation. The area of the circles is proportional to the effect of each pathway, with the color denoting the significance from the highest in red to the lowest in white.

## 3. Results

### 3.1. Clinical Data of Kidney Allografts Patients

Forty kidney transplant individuals and 20 healthy subjects were enrolled in our study to assess changes in their fecal metabolic profiles. Samples were taken at different time points from patient to patient following transplantation surgery. It is worth noting that all patients started immunosuppression therapy immediately following kidney graft (Day 0). We performed an untargeted metabolomic analysis of fecal contents using GC–MS. A clinical and demographic data investigation of several factors, including age, gender, body mass index (BMI), immunosuppressive regimen, graft period, and associated diseases, was conducted. Detailed information on the 40 stable renal allograft recipients is provided in Table 1 and supplementary Table S1. The kidney allograft recipients recruited comprised 28 men and 12 women with an average age of 42 ± 6 years and BMI of 23.7 ± 5. Overall, kidney transplant patients underwent immunosuppressive therapy involving tacrolimus (FK), mycophenolate mofetil (MMF), and steroids (Str). In the present study, 24 of the 40 patients suffered from one or multiple complications from the following list: obesity, diabetes, arterial hypertension, and dyslipidemia (AD subgroup). The remaining 16 patients were not diagnosed with any of these complications (no-AD subgroup). Primary diseases were also considered and further investigated, but they were discarded from the results reported in the present study, as they were not found as major factors to significantly highlight the shift in the fecal metabolomic profile following kidney transplantation. The control group comprised 10 men and 10 women with an average age of 44 ± 5 years and BMI of 20 ± 4. Control subjects were not undergoing any kind of treatment nor suffering from associated diseases.

### 3.2. Metabolomics Workflow

**Figure 1:**
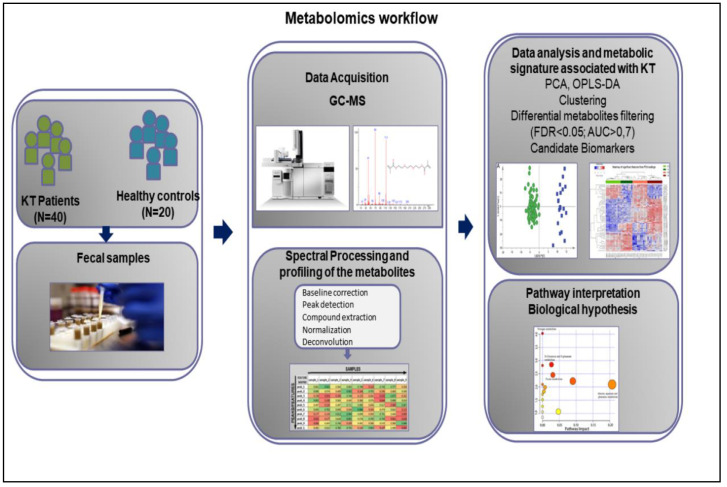
Workflow of GC–MS-based metabolomics for metabolomic profiling and data interpretation of fecal samples from stable kidney transplants patients. KT: Kidney Transplant patients; N: Number of patients and healthy subjects; PCA; Principal component analysis; OPLS-DA: orthogonal partial least squares-discriminant analysis; FDR: False Discovery Rates; AUC: Area Under the Curve.

**Figure 2:**
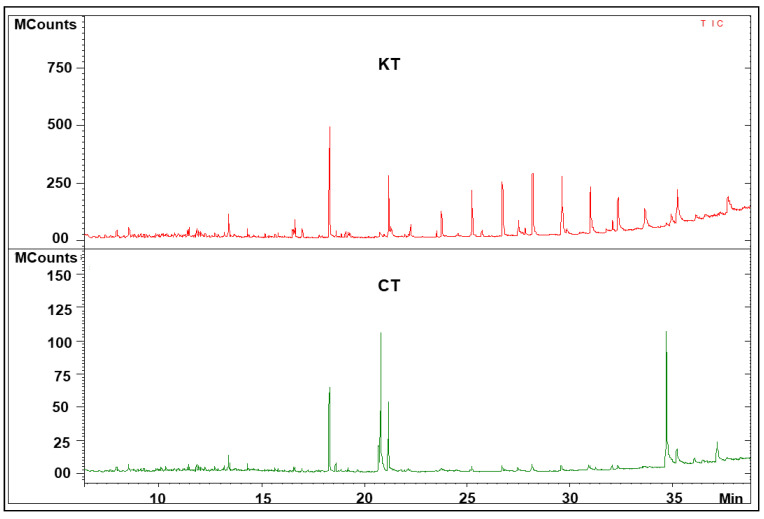
Representative fecal GC–MS spectra with retention time of samples from kidney transplant patients and healthy volunteers. **CT**: spectra from healthy controls; **KT**: spectra from kidney transplant patients.

**Figure 3:**
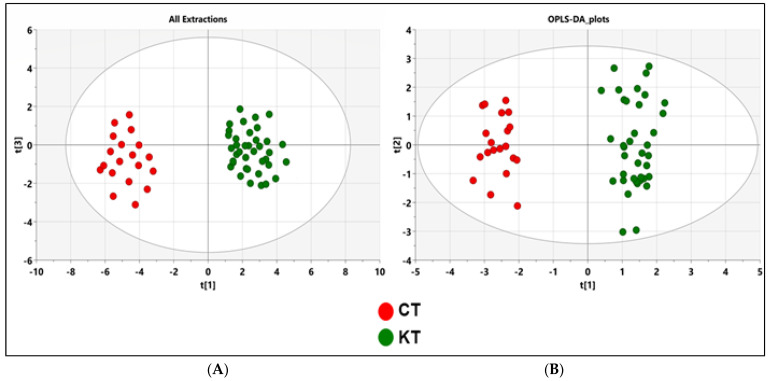
PCA and OPLS-DA score plots of the fecal metabolic profiles from the KT and control (T) groups. An overview of the data from all the five extractions confirms that there are no outlying samples within a 95% confidence interval. (**A**) PCA scores plot model with R^2^X = 0.72 and Q^2^ = 0.54. Red circles represent healthy control samples, and green circles represent KT samples. (**B**) Orthogonal partial least squares discriminant analysis (OPLS-DA) scores plot model showing separation based on all extraction methods with R^2^(X) = 0.37, R^2^(Y) = 0.958, Q^2^ = 0.841, and cross validated analysis of variance (CV-ANOVA) *p* = 1.106 × 10^−18^ values. Red circles represent healthy control samples, and green circles represent KT samples. CT: Healthy subjects group; KT: Kidney transplant patients.

**Figure 4:**
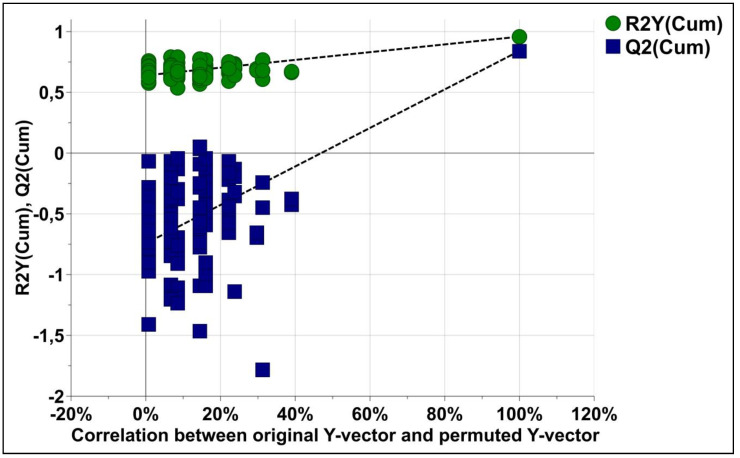
The permutation test plots for the OPLS-DA models for classification of KT and CT groups with 100 permutation tests. Green circles and blue squares represent R^2^ and Q^2^, respectively. Intercepts: R^2^ = (0.0, 0.638); Q^2^ = (0.0, −0.704).

**Figure 5:**
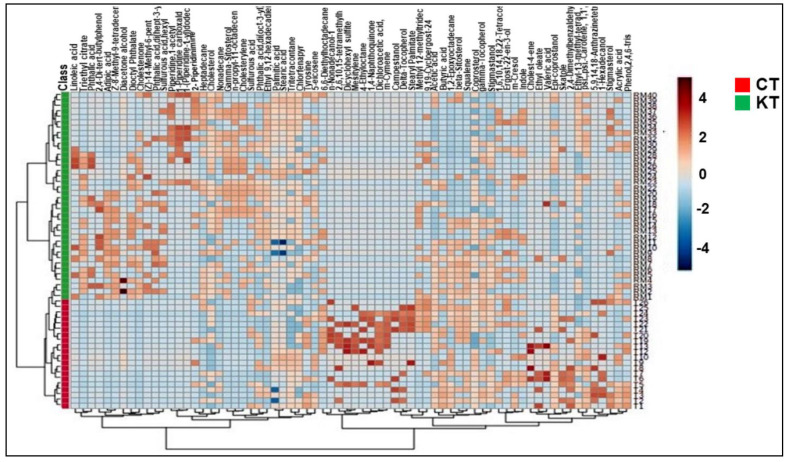
Fecal metabolic differences among groups: heat maps of differential metabolites in Table 2.

**Figure 6:**
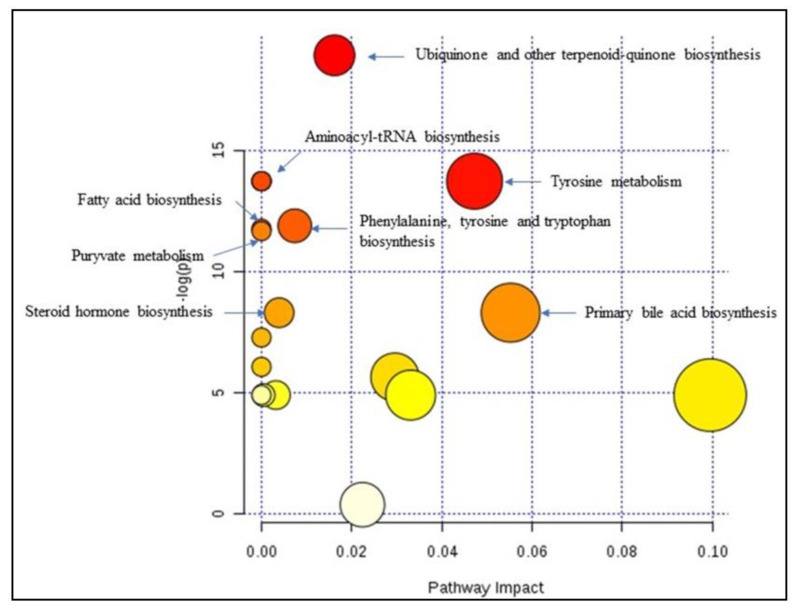
Summary of the pathway analysis with MetPA where all the metabolites were considered. The area of the bubbles is proportional to the effect of each pathway, with color denoting the significance from highest in red circles to the lowest in white circles. The red circles represents pathways with the highest *p*-value (<<0.0001), the orange circles represents pathways with *p*-value below 0.05, yellow circles represent pathways with *p*-value close to 0.05 and white circles represent pathways with *p*-value > 0.05.

Figure 1 shows the schematic workflow of our global metabolomics study. Fecal samples were collected from 40 KT patients and 20 healthy controls. Metabolites were extracted from the fecal samples following gradient of two solvent polarity and analyzed using the GC–MS approach (Figure 2). Metabolic features were extracted from each individual sample and aligned to create a metabolite-intensity table for downstream data interpretation. In total, 75 metabolic features were consistently detected in all fecal samples (Table 2) to discriminate KT patients from healthy subjects, all metabolic features were analyzed in principal component analysis (PCA) and orthogonal partial least square-discrimination analysis (OPLS-DA) (Figure 3 and Figure 4). Further, statistically significant metabolic features were extracted using the criteria of fold change *p*-value  ≤  0.05 and visualized using a heatmap (Figure 5). The potential biomarkers were further extracted using the criteria of AUC > = 0.7 and FDR  ≤  0.05 (Table 3). Metabolites were then used to construct pathway enrichment analysis to better understand their biological significance (Figure 6).

### 3.3. Analysis of Fecal Metabolic Profiling by GC–MS

Fecal samples were analyzed using a GC–MS approach that has been shown to make a comprehensive metabolic fingerprint with good analytical characteristics in fecal samples, and it was a suitable tool to investigate the metabolic abnormality following renal transplantation. In the above-described GC–MS analysis, we detected about 200 metabolites. Significant differences in the total ion chromatogram of the fecal samples were clear between renal allograft recipients and control subjects, as shown in Figure 2.

The metabolites were identified after being handled with the NIST’s three criteria: match of >800, a 90% probability of a match to NIST library standards, and a head-to-tail comparison of the fragments. After excluding the non-endogenous metabolites (such as drugs, solvents, and reagents) and those with missing values, a total of 75 metabolite features (Table 2) were detected, including both hydrophilic and hydrophobic metabolites. All 75 metabolite features were used for the following multivariate (OPLS-DA) statistical analysis.

### 3.4. Fecal Metabolic Profiles of KT Patients and Healthy Individuals Are Different

Following both ethanol and Diethyl ether extractions, the 75 metabolites were used for later statistical analysis. These included lipids (fatty acids and sterols); vitamins (Tocopherols); short-chain fatty acids; and other metabolites, such as cumenes, indoles, carotenoids, and amines. The unsupervised PCA was initially utilized on the identified peaks and the scatter plots using the score of the first principal component (PC1) and the third principal component (PC3) for each sample. As we can find, the PCA model showed a clear trend of group clustering between the kidney transplant group from the control healthy group (Figure 3A). To better show the significant fecal metabolic differences between KT and CT groups, pairwise comparative OPLS-DA was conducted with two orthogonal and one predictive component calculated for the model. The metabolomic signature showed dramatic changes in response to immunosuppressive therapy. The score plots between the CT and KT groups in the feces showed clear profile separation between both groups. The two-dimensional (2D) (Figure 3B) OPLS-DA score plots of fecal metabolite profiling among the CT and KT groups showed that the two groups could be distinguished clearly by fecal metabolite profiling with good model fitness and predictability (R^2^X = 0.37; R^2^Y = 0.958 and Q^2^ = 0.841). Furthermore, to assess differences in the metabolic structure among patients undergoing kidney graft over time, we divided our cohort into subgroups according to the post-graft period, short post-graft period (“SG”: from 3 months to 1 year; n = 11), medium-length post-graft period (“MG” from 1 year to 10 years; n = 20), and long post-transplant period (“LG” from 10 to 22 years n = 9). OPLS-DA plot showed a clear separation between healthy subjects and the associated diseases sub-groups (AD and No_AD) and showed no discrimination between the groups of patients according to their health status (supplementary Figure S2). Supplementary Figure S3 reported a total discrimination between the CT group and the different graft periods (SG, MG and LG) but no significant separation according to the post-graft period (supplementary Figure S3).

To validate the model, a permutation test with n = 100 was performed (Figure 4). Furthermore, the CV-ANOVA test was performed to check the statistical significance of the differences between the two groups in the OPLS-DA model, this resulted in a score of *p* = 1.106 × 10^−18^, indicating that the differences between the groups within the model were highly significant.

Fluctuations of the different metabolites between the two groups were investigated, and the results are exhibited in the heat maps shown in Figure 5. The relative intensity of some sterols (campestanol, coprostanol, Epi-coprostanol); fatty acids (stearic acid and linoleic acid); vitamins (gamma-tocopherol, delta-tocopherol); carotenoids (squalene), cumenes (m-cymene); alkanes (nonyl dichloroacetate); indoles (indole), an essential amino acid (tyrosine); and short-chain fatty acid (Butyric acid) were decreased in the KT patients vs. CT. However, fatty acids, the majority of sterols (cholestenone, cholesterylene, and gamma-sitosterol), alkanes (Heptadecane and 4-ethyloctate), and a carboxylic acid (Triethyl citrate) were increased in the KT patients compared to the CT group.

### 3.5. Identification of Potential Biomarkers of Kidney Transplants Status and Biological Explanation

To show potential biomarkers of kidney transplant patients and immunosuppressive therapy, relevant metabolites were selected between the control and kidney transplant groups using AUC (>0.7) and FDR (<0.05). Following both ethanol and Diethyl ether extractions, a total of 18 differential metabolites (see Table 3) in feces were chosen as potential biomarkers of kidney transplant patients.

Significantly altered metabolites (FDR < 0.05) in the feces were then used to further analyze the differential metabolic pathways in the KT patients by MetaboAnalyst v 4.0. The results revealed that the metabolic pathways of Ubiquinone and other terpenoid-quinone biosynthesis (*p* = 6.07 × 10^−9^), tyrosine metabolism (*p* = 1.09 × 10^−6^), tryptophan biosynthesis (*p* = 6.89 × 10^−5^), and primary bile acid biosynthesis (*p* = 2.49 × 10^−4^) were altered due to the impact of the immunosuppressive therapy (Figure 6). The tyrosine metabolism pathway seems to be the most significant pathway linked to the onset of kidney transplantation period and immunosuppressive therapy.

## 4. Discussion

Recently, the metabolomics approach has arisen as a promising tool to explore the metabolic profile to better assess kidney transplantation management and monitoring. Although earlier studies have been established to investigate non-targeted metabolomic assessment of serum, plasma and/or urine samples of KT subjects, reliable and consensus biomarkers are lacking.

According to our results, the fecal metabolome signature was significantly different in KT patients compared to the CT group. Furthermore, altered levels of the 18 best predictors metabolites may show significant changes in the metabolic activity of several pathways and could be associated with the immunosuppressive therapy and transplantation process. For instance, the biological interpretation of data relies first on the identification of significant metabolites and second on mapping those metabolites to biochemical pathways. Our study showed no significant discrimination between the different graft periods, and this in a total concordance with a recent study conducted by Kienana and colleagues on kidney transplant patients. In fact, they showed that the urine content was quite similar between 3 months and a year after the kidney graft [22]. Therefore, this study reported that the urinary metabolic profiles became less marked as time passed on.

Thus, there may be some possible limitations in this study. Mainly, the sample size could be the reason why it was difficult to identify significant relationships between the associated diseases as well as the primary disease factor and the kidney transplantation. Therefore, we aim to increase our cohort and study the associated pathologies one by one in order to study the association between these pathologies and kidney transplantation. Nevertheless, we intend in future studies to optimize more targeted metabolic analysis focused on specific metabolites or pathways to be able discriminate different health statuses in transplanted patients.

### 4.1. Relevant Metabolites Highlighted

KT recipients have a long history of chronic renal failure, and so, many of them suffer from lipid disorders before receiving the transplant [23]. It has been revealed that lipid classes that are relevant as biomarkers in CKD include fatty acids, glycerolipids, sphingolipids, and sterols (including steroids). After transplantation, lipid metabolism does not return to normal, even when renal function is recovered [24]. Accordingly, our results showed that fecal lipid alteration mainly increased the sterol, steroid, and fatty acid levels among KT patients. For this reason, we hypothesize that post-transplant complications, namely, dyslipidemia, hypertension, and diabetes, which are common within KT recipients, relatively correlate with lipid disorders. Steroids have been of high interest in metabolic research and kidney dysfunction, namely, the 11β hydroxysteroid dehydrogenase enzyme was found to control the bioavailability of cortisol in the kidney, which if disturbed can result in hypertension [25].

Increased serum and urinary cholesterol levels were also investigated in chronic kidney diseases because of his role in the modulation of lipid metabolism [26]. In the context of kidney transplantation, an increased cholesterol level is a risk cause for cardiovascular diseases and, therefore, has a significant impact on survival [27]. The present study is the first to assess fecal cholesterol quantification following renal graft. Herein, we found a decreased level of some fecal sterols, including campestanol, β-sitosterol, and stigmasterol. Although campestanol was not described within kidney dysfunction, low ratios of circulating β-sitosterol and stigmasterol were found by Ceglarek and co-workers [28] in line with our results. We suggest that this alteration might be associated with a high risk of allograft dysfunction and impaired clinical outcome following kidney transplantation. Nevertheless, immunosuppressive medication is reported to be a key contributor to the development of dyslipidemia in the context of kidney transplantation as well as in other solid organ transplantation (SOT), such as the heart, liver, and lungs [29]. It is well documented that an important contributor to dyslipidemia in SOT recipients is the off-target metabolic effects of immunosuppressive medications, which may alter lipoproteins and their metabolism. Lipoprotein abnormalities vary in relation to specific immunosuppressant agents and doses but typically involve increased cholesterol, as demonstrated in our study [30].

Moreover, it is believed that saturated fatty acids (SFA), mainly palmitic acid, are responsible for lipotoxicity, whereas monounsaturated fatty acids (MUFA), mainly oleic acid, have a protective effect against the apoptosis of renal cells induced by SFA [31]. In order to further highlight the potential biomarkers related to kidney health and disease, we analyzed fatty acid profiles. Our findings suggest a significant increase in linoleic acid, stearic acid, and omega-5 levels after kidney transplantation. Accordingly, Linolenic acid levels in plasma were positively associated with a change in renal function during the first year after transplantation [32], while a study by Szczuko et al. [33] found that the stearic acid level was significantly lower in the kidney transplant group compared to the control group, which was thought to suggest the activation of the pathway of palmitic acid elongation.

In recent years, non-traditional risk factors, such as inflammation and oxidative stress, have been implicated in the pathogenesis of CKD. However, the majority of studies have investigated endogenous antioxidants in predominantly advanced stages of kidney disease [34]. While the precise mechanisms remain unclear, there is substantial evidence that progressive renal decline is associated with increasing oxidative stress-altered dietary antioxidant status [35]. In this context, emerging data supports the role of carotenoids, powerful antioxidant natural compounds, in renal function and disease prevalence. In fact, a cross-sectional analysis of 570 participants showed a significant association between ca-rotenoids and a higher estimated glomerular filtration rate (eGFR) [35]. Accordingly, our results showed a decrease in these metabolites in the KT group compared to the controls. Nevertheless, our results suggested cumene as a potential biomarker that decreases in the KT group. Cumene is an endogenous metabolite belonging to terpenes, mainly found in plants, and is considered as the major constituent of essential oils extracted from plants. These bioactive natural compounds have been well described to ensure diverse biological activities, including antioxidant, anti-inflammatory, and antimicrobial. An animal study showed that cumene has antioxidant potential in vivo and may act as a neuroprotective agent in the brain [36]. Based on these promising properties, carotenoids and cumene compounds have potential clinical applications. Altogether, these new response elements may present a new strategy in the development of treatment for CDK in which oxidative stress plays an important pathophysiological role.

### 4.2. Gut Microbiota-Derived Metabolites

It has been suggested that gut microbiota could take part in the modulation of the plasma metabolome, particularly in the context of kidney disease. Yet, there are no available data on the fecal metabolome changes in kidney transplantation status. Several gut-derived metabolites relevant to kidney disease have been described, such as indoxyl sulfate, p-cresol sulfate, and trimethylamine-N-oxide (TMAO). Perhaps the most interesting advancement has been the emergence of short-chain fatty acids (SCFAs). Recent in vivo studies have demonstrated that SCFAs could modulate renal function in mice. However, the precise mechanism remains to be fully understood.

The lack of SCFAs has been demonstrated to probably contribute to uremic toxicity and local and systemic inflammation in CKD and end-stage renal disease (ESRD) [37]. Butyric acid is the most investigated SCFA in kidney diseases. In the current study, our data showed that SCFAs (butyric acid and acetic acid) have decreased within KT patients compared to CT individuals. These findings confirm the results found in our recent metagenomic study of the same cohort. In fact, we previously reported decreased abundances of *Faecalibacterium* and *Prevotella 9* genera in KT recipients after a long graft period. Most of these microorganisms are anaerobic or facultative anaerobic probiotics and produce short-chain fatty acids (SCFAs) [38]. Butyrate produced from microbial fermentation has a protective role and appears to decrease the inflammatory response [39]. Smith et al. [40] found that butyric acid regulates the differentiation of Treg cells, which plays a major role in limiting the inflammatory response. The typical butyrate-producing bacteria in CKD patients that have been identified include *Roseburia* spp. And *Faecalibacteriumprausnitzii*, as recent studies demonstrate a significant decrease in these two butyrate-producing species in CKD patients compared to healthy volunteers [41]. Furthermore, acetate is a crucial player in the inhibition of enteropathogens through *bifidobacteria* action. Damaged kidneys seem to have increased serum and urinary levels of acetate, succinate, citrate, and lactate, which are generally considered to be markers of Krebs’ cycle distress and tubular acidosis. In line with our data, Kienanaand coworkers [22] reported decreased urinary concentrations of acetate after 7 days of kidney transplantation.

Taken together, the change in SCFAs may show changes in the intestinal flora. In turn, microbiota dysbiosis could markedly impact transplantation outcomes and comorbidities.

### 4.3. Relevant Pathways Highlighted

Pathway analysis of the 26 best predictor metabolites associated with post-transplantation onset showed that the most definitely involved are Ubiquinone and other terpenoid-quinone, Phenylalanine/Tyrosine, Tryptophan, and primary bile acid pathways.

Vitamin E is best known for its role as a key player in the antioxidant defense systems in the body [42]. Our results showed a decrease in vitamin E that could be involved in the impairment of the ubiquinone pathway. Numerous studies have revealed that plasma concentrations of ubiquinone, also called coenzyme Q10 (CoQ10), are decreased in patients with chronic renal failure, suggesting a promising clinical effectiveness of CoQ10. Interestingly, an in vivo study showed that the vitamin E treatment of the chronic rat model was associated with a noticeable improvement of oxidative stress and renal dysfunction [43]. Moreover, several clinical and animal studies have recognized the beneficial potential of CoQ10 in preventing nephrotoxicity caused by the immunosuppressive treatment in kidney transplant recipients [44].

Furthermore, our results suggest that metabolic change may impact the Tyrosine and phenylalanine pathways. Several human and animal investigations have established that chronic kidney failure is associated with impairment in the metabolism or urinary excretion of two important aromatic amino acids, phenylalanine, and tyrosine. Phenylalanine is considered an essential amino acid, unlike Tyrosine (referred to as a semi-essential), which can be released from the diet, protein breakdown, and the hydroxylation of phenylalanine by phenylalanine hydroxylase through a complex mechanism [45]. A diseased kidney is marked by a reduced conversion rate of phenylalanine to tyrosine. As a result, the circulating tyrosine/phenylalanine ratio is reduced [46]. Moreover, the metabolic disorders underlying the tyrosine synthesis deficiency is highlighted by the accumulation of toxic metabolites of phenylalanine and tyrosine that actively set up a state of oxidative stress [47].

Additionally, our results showed an impairment of the tryptophan pathway. Accordingly, it has been suggested that the tryptophan pathway is involved in chronic kidney diseases. Recently, it has been demonstrated that tryptophan depletion, together with the accumulation of tryptophan-related toxic metabolites, is associated with kidney function decline and disease progression [48]. The serum and urinary levels of tryptophan and kynurenic acid have been recently used as prognostics and for monitoring the renal transplant function [49].

Bile acids are among the crucial metabolites of the intestinal microbiota. Here, fecal metabolome analysis shows, in line with earlier serum studies, a significantly increased bile acid level in kidney transplant patients undergoing immunosuppressive therapy [49]. It is currently known that the bile acid pathway is mainly involved in promoting the absorption of lipids and fat-soluble nutrients into the intestinal tract and in eliminating body cholesterol [49]. Yet, the impairment of the bile acid pathway results in the accumulation of waste intermediary metabolites that would damage several organs, such as the kidney. Nevertheless, the gut microbiota has been suggested to be a pivotal actor in this crosstalk mediating metabolic complications [50]. For these reasons, we conclude the importance of fecal analysis to help understand the bi-directional relationship between the gut microbiota and stable KT subjects revealed by a high throughput metabolomic assessment.

## 5. Conclusions

In this pioneering study, we performed an untargeted metabolomics approach to test alterations in the fecal metabolic signature of stable KT patients compared to healthy subjects. The results obtained by the GC–MS analysis of fecal samples allow us to show metabolic alterations that clearly distinguish between the studied groups. Major changes affected metabolic pathways, namely, Ubiquinone and other terpenoid-quinone, Phenylalanine/Tyrosine, Tryptophan, and primary bile acid pathways, which have been previously discussed within kidney disease outcomes. Nevertheless, this pilot study indicates that there is no difference in endogenous metabolites between long-term and short-term post-transplant patients. This may strengthen the relevance of the proposed markers. Overall, the current results seem to be an interesting matter for further investigation that may contribute to the discovery of promising biomarkers applicable to the sensitive monitoring of kidney transplantation and the development of new therapeutic approaches.

## Figures and Tables

**Table 1:** Clinical and demographic data of study groups.

Subjects	Age (Y) Mean (SD)	Gender	Diet	BMI	Immunosuppressive Therapy	Period (Y) After Tx Mean (SD)
Patients	42 (6)	28 M/12 F	Low Salt	23.7 (5)	Str/Fk/MMF	6 (5)
Controls	44 (5)	10 M/10 F	Balanced	20 (4)	-	-

**Y**: year; **SD**: Standard deviation; Str: steroids; FK: tacrolimus; MMF: mycophenolic acid; (-): not applicable; F: female; M: male; **Tx**: treatment.

**Table 2:** Summary of the detected metabolites in the feces of KT and CT group.

Chemical Classes	Compounds	m/z	Chemical Structure
Alkanes	Tritetracontane	57.99 / 71.64 /43.47	MF: C43H88 MW: 605.2 g/mol
	1,6,10,14,18,22-Tetracosahexaen-3-ol, 2,6,10,15,19,23-hexamethyl	96/81/41	MF: C30H50O MW: 426.7 g/mol
	6,6-Diethylhoctadecane	57/71/85	MF: C22H46 MW: 310.6 g/mol
	nonadecane	57.99 / 43.82 / 71.66	MF: C19H40 MW: 268.5 g/mol
	Heptadecane	57.99 / 43.65 / 71.64	MF: C17H36 MW:240.5 g/mol
	4-Ethyloctane	57/43/41	MF: C10H22 MW: 142.28 g/mol
	1,2-Epoxyoctadecane	82/55/71	MF: C18H36O MW: 268.5 g/mol
Amines	1-Piperidine carboxaldehyde	113.08/ 56.05/ 84.04	MF: C6H11NO MW: 113.16 g/mol
	Piperidine,1-acetyl	84/43/127	MF: C7H13NO MW: 127.18 g/mol
	1-(Piperidine-1-yl)dodecan-1-ol	127.22/140.85/84.14	MF: C17H33NO MW: 267.4 g/mol
Alkaloids	2- Piperidinimine	98.06/ 70.55/43.78	MF: C5H10N2 MW: 98.15 g/mol
Amino Acid	Tyrosine	218.18/ 71.85/ 43.41	MF: C9H11NO3 MW: 181.19 g/mol
Benzenoids	Phthalic acid,di(oct-3-yl) ester	149/ 167/ 150	MF: C24H38O4 MW: 390.6 g/mol
	2,4-Dimethylbenzaldehyde	134.07/ 133.06/ 105.06	MF: C9H10O MW: 134.17 g/mol
	Dioctyl Phthalate	149.99/ 391.66/ 261.40	MF: C24H38O4 MW: 390.6 g/mol
	Phthalic acid,di(hept-3-yl)	149.02/ 167.03/ 150	MF: C22H34O4 MW: 362.5 g/mol
	Phthalic acid	149.1/ 139.2/ 121.3	MF: C8H6O4 MW: 166.13 g/mol
	Mesitylene	105.99/ 120.86/77.98	MF: C9H12 MW: 120.19 g/mol
Carboxylic Acids	Acrylic acid	72.2/27.23/55.17	MF:C3H4O2 MW: 72.06 g/mol
	Triethyl Citrate	157/ 203/115	MF: C12H20O7 MW: 276.28 g/mol
Carotenoids	Squalene	69.06/81.06/41.03	MF: C30H50 MW: 410.7 g/mol
	psi.,.psi.-Carotene, 1,1′,2,2′-tetrahydro-1,1′-bis[(trimethylsilyl)oxy]	73.99/ 69.53/ 91.51	MF: C42H64O2 MW: 600.49 g/mol
Cumenes	m-Cymene	93.02/ 135.11/ 121.08	MF: C10H14 MW: 134.22 g/mol
Dialkyldisulfides	Dicyclohexyl sulfite	83/55/41	MF: C12H22O3S MW: 246.37 g/mol
Dicarboxylic Acids	Di2-ethylhexyladipate	129/371/259	MF: C22H42O4 MW: 370.6 g/mol
Long-Chain Fatty Acids	Methyl 12-methyltridecanoate	74/55/75	MF: C15H30O2 MW: 242.4 g/mol
	Stearic acid	117/129/132	MF: C18H36O2 MW: 284.5 g/mol
	Palmitic acid	237.3/255.3/227.1	MF: C16H32O2 MW: 256.42 g/mol
	Linoleic acid	263.1/256.1/95	MF: C18H32O2 MW: 280.4 g/mol
Short Chain Fatty Acids	Acetic acid	43/55/60	MF: C2H4O2 MW: 60.05 g/mol
	Butyric acid	43.05/87.04/29.04	MF: C4H8O2 MW: 88.11 g/mol
Fatty Acids	Adipic acid	129.1/115.1/119.2	MF: C6H10O4 MW: 146.14 g/mol
	Dichloroacetic acid, 4-hexadecyl ester	55.99/69.79/83.76	MF: C18H34Cl2O2 MW: 352.19 g/mol
	Valeric acid	60.02/29.04/27.03	MF: C5H10O2 MW: 102.13 g/mol
	Z-8-Methyl-9-tetradecenoic acid	55/41/43	MF: C15H28O2 MW: 240.38 g/mol
	Ethyl-14-methyl-hexadecanoate	88/55/57	MF: C19H38O2 MW: 298.5 g/mol
Fatty Acids Esters	Ethyl oleate	43.99/55.68/69.67	MF: C20H38O2 MW: 310.29 g/mol
	Ethyl 9,12-hexadecadienoate	67/81/55	MF: C18H32O2 MW: 280.4 g/mol
	Ethyl-13-methyl-tetradecanoate	88/101/55	MF: C17H34O2 MW: 270.5 g/mol
	Ethyl hexadecanoate	257.5/219.3/237.3	MF: C18H36O2 MW: 284.5 g/mol
	n-propyl-11-octadecenoate	55/69/83	MF: C21H40O2 MW: 324.5 g/mol
	Stearyl Palmitate	257/57/43	MF: C34H68O2 MW: 508.9 g/mol
Fatty Alcohol	1-Hexadecanol	55.99/69.82/83.76	MF: C16H34O MW: 242.26 g/mol
	n-Nonadecanol-1	83/55/97	MF: C19H40O MW: 284.5 g/mol
Fatty Aldehydes	(Z)-14-Methyl-6-pentadecenoic acid	73.05/89.04/43.7	MF: C16H30O2 MW: 254.41 g/mol
Hydrocarbons	5-eicosene	55.99/57.77/43.69	MF: C20H40 MW: 280.31 g/mol
Indoles	Indole	117.05/89.02/101.03	MF: C8H7N MW: 117.15 g/mol
	Skatole	131.07/103.04/77.03	MF: C9H9N MW: 131.17 g/mol
Ketones	Diacetone alcohol	43.02/59.05/57.03	MF: C6H12O2 MW: 116.16 g/mol
Non-metal sulfates	Sulfurous acid,hexyl octyl ester	57/85/43	MF: C14H30O3S MW: 278.45 g/mol
	Sulfurous acid	81.97/53.9/64.96	MF: H2SO3 MW: 82.08 g/mol
Not Attributed	3,9.beta:14,15-Diepoxypregn-16-en-20-one,3,11.beta.,18-triacetoxy-	429.98/43.67/430.73	MF: C27H34O9 MW: 502.6 g/mol
	Alanine, N-methyl-n-propoxycarbonyl-, isohexyl ester	144/102/43	MF: C14H27NO4 MW: 273.37 g/mol
	5,9,14,18-Anthrazinetetrone, 6,15-dihydro-8-hydroxy-	458.09/49.55	MF: C28H14N2O5 MW: 458.4 g/mol
Phenols	m-Cresol	81.03/91.05/67.01	MF: C7H8O MW: 108.14 g/mol
	Phenol,2,4,6-tris(1-methylethyl)	205.78/220.12/206.34	MF: C15H24O MW: 220.35 g/mol
Phenylpropanes	2,4-Di-tert-butylphenol	191.99/57.32/41.16	MF: C14H22O MW: 206.17 g/mol
Prenol lipids	2,6,10,15-tetramethylheptadecane	57.06/71.09/43.99	MF: C21H44 MW: 296.6 g/mol
Quinones	1,4-Naphthoquinone,6-acetyl	217.99/232.91/189.44/43.27	MF: C12H8O5 MW: 232.04 g/mol
Sterols	Coprosterol	388.32/44.05/233.15	MF: C27H48O MW: 388.7 g/mol
	beta-Sitosterol	43.99/ 55.35/41.33	MF: C29H50O MW: 414.39 g/mol
	Cholesterol	43.99/55.89/57. 74	MF: C27H46O MW: 386.35 g/mol
	Ergost-22-en-3-ol (3alpha,5beta,22E)	55.04/69.06/81.04	MF: C28H48O MW: 400.5 g/mol
	Stigmastanol	43.99/107.71/215.7	MF: C29H52O MW: 416.4 g/mol
	Cholestenone	124/43/55	MF: C27H44O MW: 384.6 g/mol
	Gamma-Sitosterol	43/55/41	MF: C29H50O MW: 414.7 g/mol
	4,6-cholestadienol	43.99/143.7/135.63	MF: C27H44O MW: 384.34 g/mol
	Stigmasterol	255/83/159	MF: C29H48O MW: 412.7 g/mol
	Cholesterylene	368/81/147	MF: C27H44 MW:368.6g/mol
Steroids	Epi-coprostanol	215/55/43	MF: C27H48O MW: 388.7 g/mol
	Campestanol	215/233/234	MF: C28H50O MW: 402.7 g/mol
	Cholest-4-ene	370.99/108.92/43.39	MF: C27H46 MW: 370.36 g/mol
Stanol	9,19-Cycloergost-24(28)-en-3-ol,4,14-dimethyl-, (3β,4α,5α)-	412.36/369.33/43.05	MF: C30H50O MW:426.71 g/mol
Tocopherols	gamma-Tocopherol	151.99/416.72/ 417.21	MF:C28H48O2 MW: 416.37 g/mol
	Delta-Tocopherol	402/138/177	MF: C27H46O2 MW: 402.7 g/mol

MF: molecular formula; MW: molecular weight.

**Table 3:** Biomarkers identified in fecal metabolic profiles of KT group vs. CT group. ^a^ Area under the receiver operating characteristic (ROC) curve of the biomarkers; ^b^ sensitivity and ^c^ specificity were calculated from the ROC curve.

Compound	FDR	FC	AUC ^a^	Sensitivity ^b^	Specificity ^c^	Pathways
Cholestenone	0.00168	1.7408 × 10^−5^	0.86579	0.77	0.951	Steroid degradation
Triethyl Citrate	0.00147	4.4656 × 10^−6^	0.84013	0.725	0.921	Not identified
Gamma-Sitosterol	0.0161	3.4307 × 10^−5^	0.81711	0.709	0.916	Not identified
Cholesterylene	0.0153	2.3588	0.79868	0.684	0.906	Steroid hormone biosynthesis
Ethyl 9,12-hexadecadienoate	0.0133	1.0012 × 10^−5^	0.78158	0.654	0.906	Primary bile acid biosynthesis; Steroid hormone biosynthesis
Adipic acid	0.00158	21.854	0.77961	0.649	0.891	Caprolactam degradation
Nonyl dichloroacetate	0.00769	9.9808 × 10^−6^	0.76974	0.657	0.876	Not identified
Dioctyl Phthalate	0.0022	9.0143 × 10^−6^	0.76908	0.641	0.875	Not identified
Delta-Tocopherol	0.0131	20654.0	0.75395	0.607	0.896	Ubiquinone and other terpenoid-quinone biosynthesis
Cholesterol	0.00675	4.46284	0.74803	0.609	0.866	Steroid hormone biosynthesis; Primary bile acid biosynthesis
Tyrosine	0.00985	57.844	0.74408	0.619	0.859	Ubiquinone and other terpenoid-quinone biosynthesis; Tyrosinemetabolism, Phenylalanine, tyrosine and tryptophan biosynthesis; Aminoacyl-tRNA biosynthesis
m-cymene	0.036354	105790.0	0.74276	0.699	0.866	Not identified
Indole	0.04437	45722.0	0.72763	0.671	0.87	Phenylalanine, tyrosine and tryptophan biosynthesis
Epi-coprostanol	0.0337	10665.0	0.71645	0.685	0.838	Not identified
nonadecane	0.0118	0.29481	0.71645	0.599	0.845	Not identified
Sulfurous acid,hexyl octyl ester	0.00715	0.12937	0.7125	0.566	0.831	Cysteine and methionine metabolism
gamma-tocopherol	0.00421	2.1326	0.71118	0.644	0.778	Ubiquinone and other terpenoid-quinone biosynthesis
Stigmasterol	0.02382	6.593 × 10^−5^	0.70724	0.656	0.764	Steroid biosynthesis

**FC**: fold change; **FDR**: false discovery rates; **AUC**: Area Under the Curve.

## Data Availability

A data available on request by the First author; Soumaya Kouidhi (soumayakouidhi@gmail.com).

## Supplementary Materials

The following are available online at https://www.mdpi.com/article/10.3390/diagnostics11050807/s1, Table S1: Clinical and demographic data of kidney transplantat patients. Figure S1: Quality control of system suitability for GC–MS metabolomics using two pesticide compounds: A: the curve calibration of malathion with r^2^ = 0.950 and B: the curve calibration of chlorpyrifos with r^2^ = 0.968. Figure S2: OPLS-DA score plots of the fecal metabolic profiles from the KT sub-groups with associated diseases (AD) and without associated disease no-AD compared to healthy subjects: OPLS-DA scores plot model showing no separation between the two groups based on all extraction methods with R2(X) = 0.617, R2(Y) = 0.507, Q2 = 0.331, and cross validated analysis of variance (CV-ANOVA) *p* = 0.89. Red circles represent the CT group; Blue circles represent AD patients and Green circles represent No-AD patients, Figure S3: OPLS-DA score plots of the fecal metabolic profiles from the KT sub-groups with different graft periods: OPLS-DA scores plot model showing no discrimination between the different groups based on all extraction methods with R2(X) = 0.358, R2(Y) = 0.471, Q2 = 0.293 and cross validated analysis of variance (CV-ANOVA) *p* = 0.65. Red circles represent the CT group; blue circles represent patients with short period graft (SG); green circles represent patients with long period graft (LG); and yellow circles represent patients with medium period graft (MG).
