# Supplementary material for: Fecal Metabolomics Reveals Distinct Profiles of Kidney Transplant Recipients and Healthy Controls
Source: Diagnostics (Basel). 2021 Apr 29;11(5):807. doi: 10.3390/diagnostics11050807 (PMC8145417; doi:10.3390/diagnostics11050807)

Supplementary Table S1: Clinical and demographic data of Kidney transplanted patients

Patient	Age	Sex	PD	Immunosuppression	Months after Tx	Associated Diseases	DD (mg)	ATH	LLM	BMI
**P1**	21	M	Ind N	Str/Rapa	12	-	2	-	-	18,04
**P2**	53	M	CN	Str/FK	156	T2D/HBP	1	-	-	29,7
**P3**	35	F	TIN	FK /AZA	120	-	-	-	-	18,3
**P4**	47	M	Ind N	Str/CsA /MMF	60	HU/HBP	1	-	-	31,7
**P5**	67	M	CRAN	Str/FK /MMF	264	HU/HBP/DysLP	1	CEI	-	26,5
**P6**	37	M	-	Str/FK /MMF	84	HU/HBP/NCA	1	CEI/CCB	CLS	28,08
**P7**	37	M	CRAN	FK /MMF	60	-	4	CEI	-	26,9
**P8**	66	M	VN	Str/FK /MMF	72	HBP/H Str	4	CCB	-	26,3
**P9**	48	F	Ind N	Str/FK /AZA	96	-	-	-	-	16,13
**P10**	18	F	CTIN	Str/FK	24	Car S	4	-	-	19,2
**P11**	29	M	Ind N	Str/FK /MMF	144	-	2	-	-	15,8
**P12**	58	M	IN	Str/FK /MMF	96	HU/HBP	5	CCB/BB	-	24,48
**P13**	33	F	SLE	Str/FK /MMF	12	DysLP	-	-	-	19,9
**P14**	56	M	Ind N	Str/FK /MMF	72	T2D/HBP/NCA/DysLP	-	CEI/CCB/BB	CLS	30,8
**P15**	39	M	-	Str/FK /MMF	48	T2D/HBP/DysLP	-	CEI/CCB	LovaCLS	27,5
**P16**	57	M	CRAN	Str/FK /AZA	72	DysLP	6	CEI	Lip	23,5
**P17**	53	M	Ind N	Str/FK /MMF	84	T2D/HBP/NCA	-	CEI/CCB	CLS	32,5
**P18**	51	M	DN	Str/CsA /MMF	84	T2D/HBP/DysLP	225	CCB/BB	Lip	24,5
**P19**	46	M	Ind N	Str/FK /MMF	96	DysLP	3	CEI/CCB	CLS	24,2
**P20**	36	F	Ind N	Str/FK /MMF	156	HBP/DysLP	5	CEI/CCB/BB	Lip	-
**P21**	60	F	CGN	Str/FK /AZA	60	T2D/DysLP	-	-	-	37,7
**P22**	17	M	TIN	Str/FK /MMF	108	-	3	-	Lip	20,7
**P23**	26	M	-	Str/FK /MMF	12	-	-	-	-	20,3
**P24**	42	F	CGN	Str/FK /AZA	36	-	-	-	-	32
**P25**	43	F	CGN	Str/CsA /AZA	168	-	-	-	-	29,9
**P26**	42	F	Ind N	Str/FK /MMF	-	HBP	-	-	-	25,5
**P27**	50	F	SLE	Str/FK /MMF	120	-	5	CCB/BB	-	32,6
**P28**	41	M	Ind N	Str/FK /MMF	36	SNHL	-	CEI/CCB	-	24,6
**P29**	27	M	Ind N	FK /AZA	96	NCA	-	CEI	-	19,9
**P30**	61	M	-	Str/CsA /MMF	216	HBP/CAD	-	CEI/CCB/BB	-	27,3
**P31**	43	F	Ind N	Str/FK /AZA	144	HU	-	-	CLS	-
**P32**	37	M	TIN	Str/FK /MMF	8	HBP	-	CCB/BB	-	21,3
**P33**	30	M	Ind N	Str/FK /MMF	3	T2D/DysLP	-	-	-	-
**P34**	50	M	VN	Str/FK /MMF	84	T2D/HBP/DysLP	-	CCB/BB	Lip	27,16
**P35**	41	M	GN	Str/FK /MMF	132	T2D/DysLP	-	-	CLS	-
**P36**	30	M	Ind N	Str/Rapa	7	-	-	-	CLS	-
**P37**	39	M	Ind N	Str/FK /MMF	10	DysLP	-	-	-	23,18
**P38**	47	M	IN	Str/CsA /MMF	216	H Str	225	-	Lip	26,5
**P39**	42	F	-	Str/FK /MMF	120	HBP/NCA	-	CEI/CCB	-	25,4
**P40**	44	M	VN	Str/FK /MMF	108	HBP/DysLP	-	CEI/CCB	-	23,6

PD= Ind N: Inditerminate Nephropathy; CGN: chronic glomerulonephritis; GN: glomerulonephritis; SLE: Lupus nephritis ; TIN: tubulointerstitial Nephritis; CTIN: chronic tubulointerstitial Nephritis; IN: Interstitial Nephritis; CN: chronic nephropathy; CRAN: chronic renal allograft nephropathy; VN: vascular nephropathy

Immunosuppression= Str: steroids Rapa:Rapamicin (Sirolimus); FK: Tacrolimus ; CsA:cyclosporin; MMF: mecophenolate mofetil ; AZA: Azathioprine

AD= T2D: type 2 Diabetes; HPB: hyper blood pressure; HU: hyperuricemia; DysLP: Dyslipidemia; HStr: Hemorragic stroke; SNHL: Sensorineural hearing loss; NCA: Neurocirculatory asthenia; CAD: coronary artery disease

DD= Daily dose of (CsA, FK, Rapa)

AHT= Antihypertensives ;CEI: angiotensin-converting enzyme inhibitor ; CCB: calcium channel blockers; BB: Beta blockers

LLM= Lipid lowering medications; CLS: statin; LovaCLS: lovastatin; LIP: lipanthyl

Figure S1

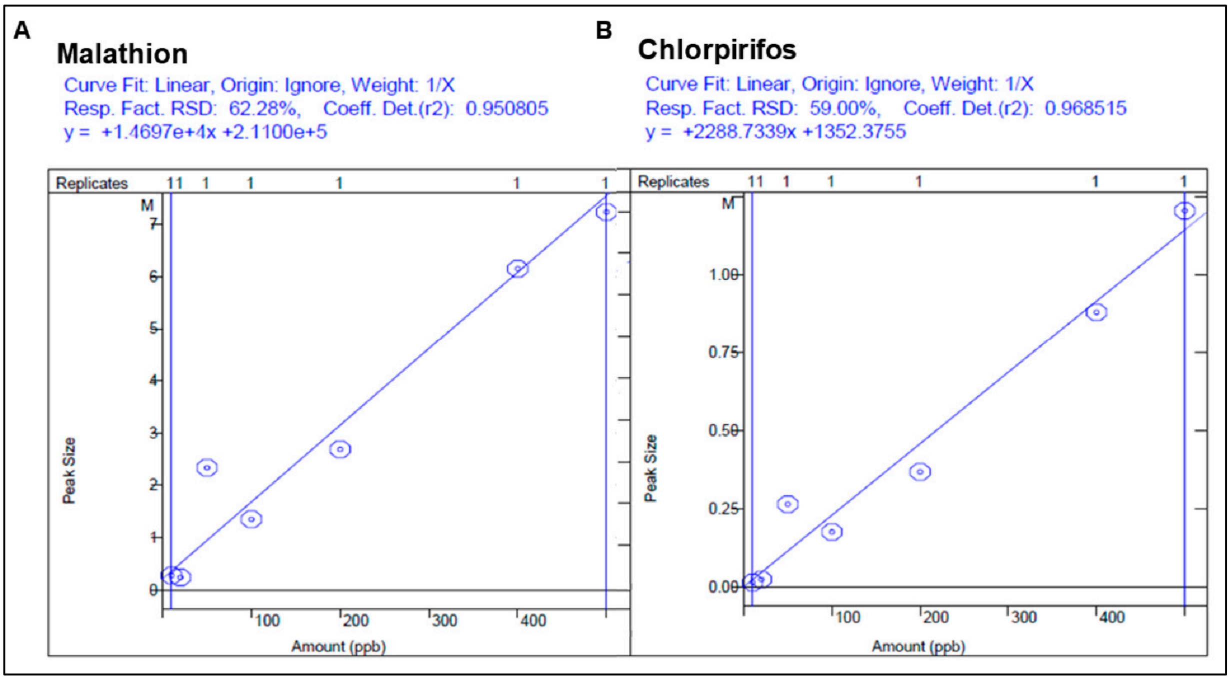

Figure S2

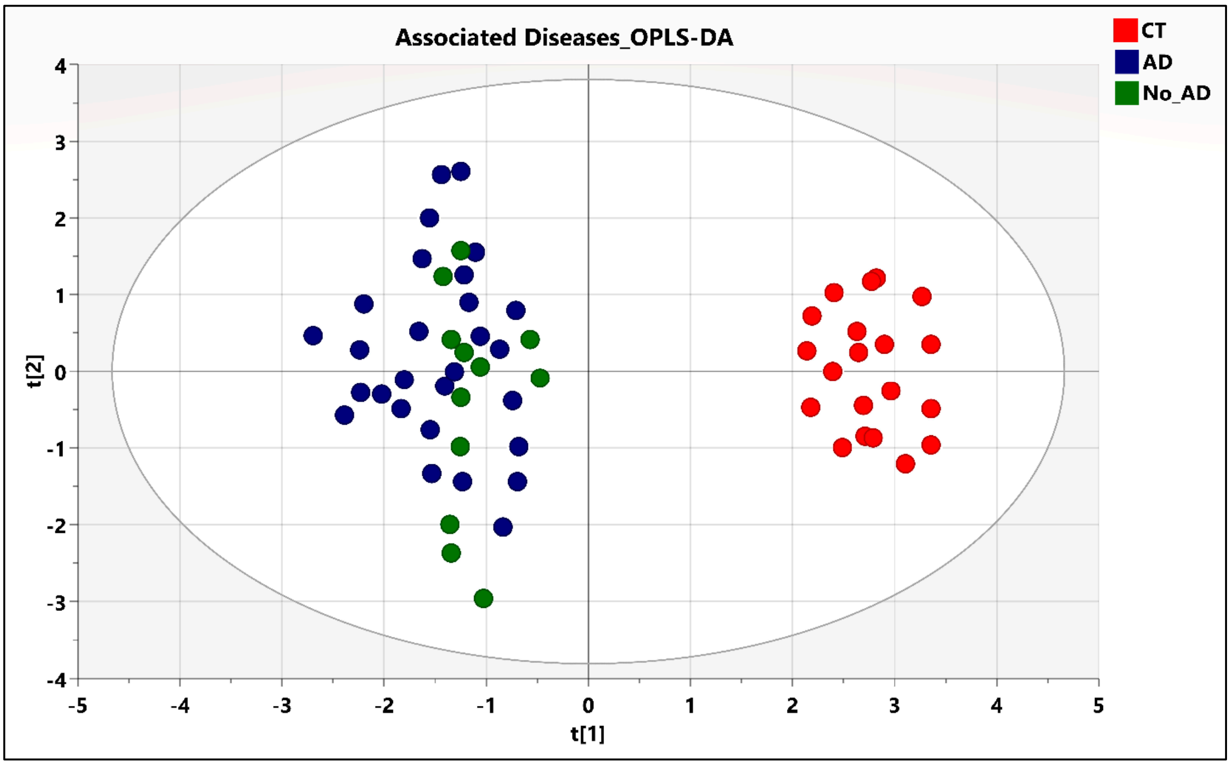

Associated Diseases_OPLS-DA
CT
AD
No_AD
4
3
2
1
0
-1
-2
-3
-4
t[2]
-5
-4
-3
-2
-1
0
1
2
3
4
5
t[1]

Figure S3

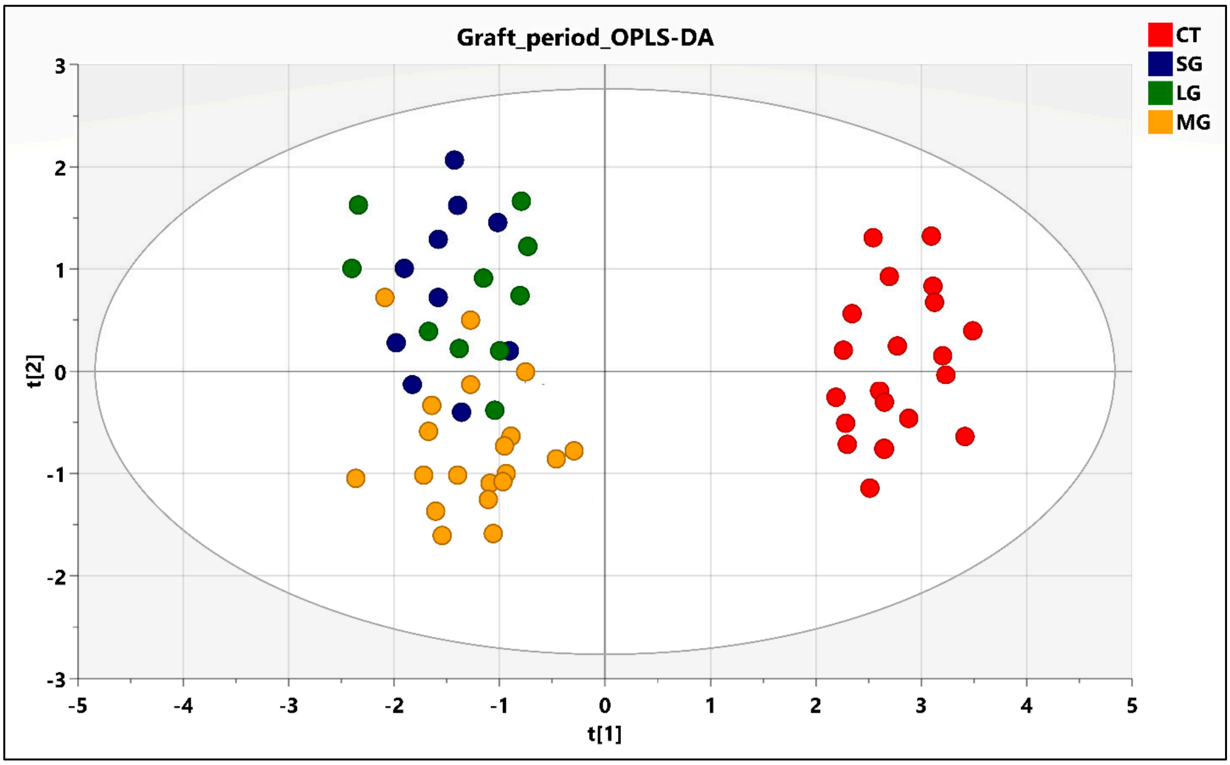

Supplement: Supplementary file 1 [file diagnostics-11-00807-s001.zip › diagnostics-1158918-supplementary.pdf]
